# First-Principles Assessment of CdTe as a Tunnel Barrier at the α-Sn/InSb Interface

**DOI:** 10.1021/acsami.3c00323

**Published:** 2023-03-20

**Authors:** Malcolm J. A. Jardine, Derek Dardzinski, Maituo Yu, Amrita Purkayastha, An-Hsi Chen, Yu-Hao Chang, Aaron Engel, Vladimir N. Strocov, Moïra Hocevar, Chris Palmstro̷m, Sergey M. Frolov, Noa Marom

**Affiliations:** † Department of Physics and Astronomy, University of Pittsburgh, Pittsburgh, Pennsylvania 15260, United States; ¶ Department of Materials Science and Engineering, Carnegie Mellon University, Pittsburgh, Pennsylvania 15213, United States; § Department of Physics and Astronomy, University of Pittsburgh, Pittsburgh, Pennsylvania 15260, United States; ∥ Université Grenoble Alpes, CNRS, Grenoble INP, Institut Néel, Grenoble 38000, France; ⊥ Materials Department, University of California-Santa Barbara, Santa Barbara, California 93106, United States; # Paul Scherrer Institut, Swiss Light Source, Villigen PSI CH-5232, Switzerland; ○ Department of Electrical and Computer Engineering, University of California-Santa Barbara, Santa Barbara, California 93106, United States; △ Department of Chemistry, Carnegie Mellon University, Pittsburgh, Pennsylvania 15213, United States

**Keywords:** density functional theory, electronic structure, angle resolved photoemission spectroscopy, metal−semiconductor interface, tunnel barrier, InSb, CdTe, Sn

## Abstract

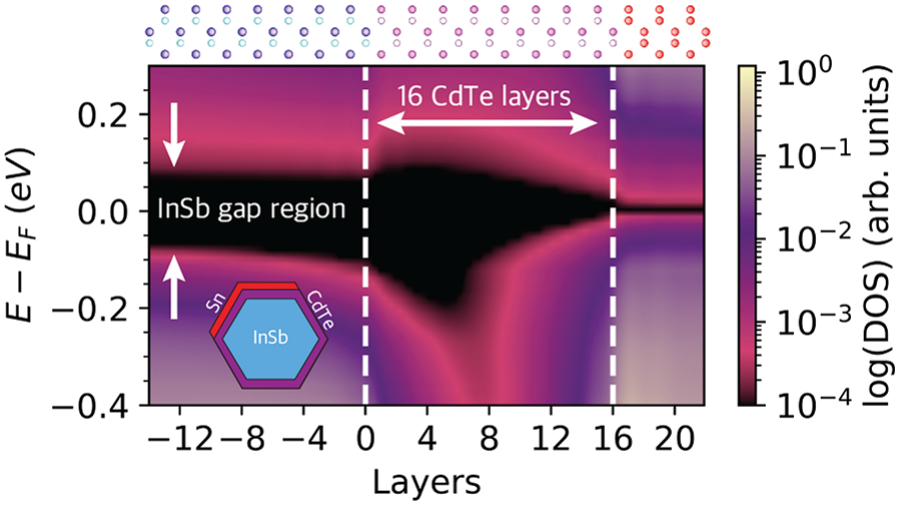

Majorana zero modes, with prospective applications in topological quantum computing, are expected to arise in superconductor/semiconductor interfaces, such as β-Sn and InSb. However, proximity to the superconductor may also adversely affect the semiconductor’s local properties. A tunnel barrier inserted at the interface could resolve this issue. We assess the wide band gap semiconductor, CdTe, as a candidate material to mediate the coupling at the lattice-matched interface between α-Sn and InSb. To this end, we use density functional theory (DFT) with Hubbard U corrections, whose values are machine-learned via Bayesian optimization (BO) [npj Computational Materials2020, 6, 18010.1038/s41524-020-0337-2PMC1157487239563780]. The results of DFT+U(BO) are validated against angle resolved photoemission spectroscopy (ARPES) experiments for α-Sn and CdTe. For CdTe, the *z*-unfolding method [Advanced Quantum Technologies2022, 5, 2100033] is used to resolve the contributions of different *k*_*z*_ values to the ARPES. We then study the band offsets and the penetration depth of metal-induced gap states (MIGS) in bilayer interfaces of InSb/α-Sn, InSb/CdTe, and CdTe/α-Sn, as well as in trilayer interfaces of InSb/CdTe/α-Sn with increasing thickness of CdTe. We find that 16 atomic layers (3.5 nm) of CdTe can serve as a tunnel barrier, effectively shielding the InSb from MIGS from the α-Sn. This may guide the choice of dimensions of the CdTe barrier to mediate the coupling in semiconductor–superconductor devices in future Majorana zero modes experiments.

## Introduction

A promising route toward the realization of fault-tolerant quantum computing schemes is developing materials systems that can host topologically protected Majorana zero modes (MZMs).^1−3^ MZMs may appear in one-dimensional topological superconductors,^4−7^ which can be effectively realized by proximity coupling a conventional superconductor and a semiconductor nanowire that possesses strong spin–orbit coupling (SOC). Adding in a magnetic field enables this system to behave as an effective spinless p-wave topological superconductor, which allows for MZM states. Recently, there have been new developments in material choices and experimental methods to identify MZMs in semiconductor nanowire–superconductor systems, designed to overcome challenges identified during the first wave of experiments.^8−10^ These include trying new combinations of semiconductors and epitaxial superconductors, e.g., Pb, Sn, and Nb, to maximize the electron mobility and utilize larger superconducting gaps and higher critical magnetic fields.^11−15^ Additionally, new proposed architectures include creating nanowire networks and inducing the field via micromagnets.^16,17^

One of the challenges presented by the superconductor/semiconductor nanowire construct, is that excessive coupling between the superconducting metal and semiconductor may “metallize” the semiconductor, thus rendering the topological phase out of reach. Theoretical studies that treated the semiconducting and superconducting properties via the Poisson–Schrödinger equation have shown that excessive coupling between the materials may lead to the semiconductor’s requisite properties, such as the Landé *g*-factor and spin–orbit-coupling (SOC), being renormalized to a value closer to the metal’s. In addition, large unwanted band shifts may be induced.^12,18−25^ Having a tunnel barrier could modulate the superconductor–semiconductor coupling strength and thus the induced proximity effect, which is critical for controlling experiments. It is currently unknown what the required width range of a tunnel barrier would be.

InSb and Sn are among the materials used to fabricate devices for Majorana research.^26^ InSb is the backbone of such systems because it has the highest electron mobility, strongest spin–orbit coupling (SOC) and a large Landé *g*-factor in the conduction band compared to other III–V semiconductors. β-Sn has a bulk critical field of 30 mT and a superconducting critical temperature of 3.7 K, higher than the 10 mT and 1 K, respectively, of Al. Recently, β-Sn shells have been grown on InSb nanowires, inducing a hard superconducting gap.^12^ The large band gap semiconductor CdTe is a promising candidate to serve as a tunnel barrier. Thanks to its relative inertness, it may simultaneously act as a passivation layer protecting the InSb from environmental effects and potentially minimizing disorder.^27,28^ Advantageously, CdTe is lattice matched to InSb.^29^ Sn has two allotropes. The β form, with a BCT crystal structure, is of direct relevance to MZM experiments thanks to its superconducting properties. However, the semimetallic α form has a diamond structure, which is lattice matched to InSb and CdTe, making it an ideal model system for investigating, both theoretically and experimentally, the electronic structure of Sn/InSb heterostructures.

Much experimental work, such as growth and ARPES studies, has been undertaken on α-Sn. Previously, α-Sn has been found to possess a topologically trivial band inversion, with SOC inducing a second band inversion and a topological surface state (TSS).^30,31^ The effect of strain on the topological properties of α-Sn has also been studied.^20,32−41^ In-plane compressive strain has been reported to make α-Sn a topological Dirac-semimetal and induce a second TSS to appear.^30^ Conversely, tensile strain has been reported to induce a transition to a topological insulator. CdTe^28^ and α-Sn^12,31^ have been epitaxially grown on InSb. Depositing Sn on InSb often leads to growth of epitaxially matched α-Sn, although β-Sn may appear under some conditions.^42^ In addition, α-Sn can transition to β-Sn if the Sn layer is above a critical thickness or if heat is applied during the fabrication process.^43,44^ Studying the interface with the lattice matched α-Sn may provide insight, which is also pertinent to β-Sn, as both could be present in hybrid systems. Therefore, these are promising materials to investigate for future device construction.

MZM experiments rely on finely tuned proximity coupling between a superconducting metal and a semiconductor. By adding a tunnel barrier at the interface between the two materials and varying its width, one could potentially modulate the proximity coupling strength to achieve precise control over the interface transparency. To the best of our knowledge, this idea has not yet been tested in experiments, and it is presently unknown which material(s) would be the best choice for a barrier and what would be the optimal thickness. Simulations of a trilayer system with a tunnel barrier are therefore needed to inform MZM experiments. Here, we use density functional theory (DFT) to study a trilayer system, in which InSb is separated from α-Sn by a CdTe tunnel barrier. Despite recent progress toward treating superconductivity within the framework of DFT,^45,46^ the description of proximity-induced superconductivity at an interface with a semiconductor is still outside the reach of present-day methods. However, DFT can provide useful information on properties, such as the band alignment at the interface. Conduction band offsets are of particular importance because the proximity effect in most experiments on InSb primarily concerns the conduction band. In addition, DFT can provide information on the penetration depth of metal induced gap states (MIGS) into the semiconductor.^19,28,47,48^

Within DFT, computationally efficient (semi)local exchange-correlation functionals severely underestimate the band gap of semiconductors to the extent that some narrow-gap semiconductors, such as InSb, are erroneously predicted to be metallic.^49^ This is attributed to the self-interaction error (SIE), a spurious repulsion of an electron from its own charge density.^50^ Hybrid functionals, which include a fraction of exact (Fock) exchange, mitigate the SIE and yield band gaps in better agreement with experiment. However, their computational cost is too high for simulations of large interface systems, such as the α-Sn/CdTe/InSb trilayer system studied here. The DFT+U approach, whereby a Hubbard U correction is added to certain atomic orbitals, provides a good balance between accuracy and computational cost.^49,51^ Recently, some of us have proposed a method of machine learning the U parameter for a given material by Bayesian optimization (BO).^52^ The DFT+U(BO) method has been employed successfully for InSb and CdTe.^53^

It has been shown that (semi)local functionals fail to describe the bulk band structure of α-Sn correctly, specifically the band ordering and the orbital composition of the valence bands at the Γ point. DFT+U, hybrid functionals, or many-body perturbation theory within the GW approximation yield a correct description of the band structure.^32,38,54−56^ DFT+U simulations have required slab models of more than 30 monolayers of Sn to converge toward a bulk regime, where quantum confinement is no longer dominant. With a small number of layers, α-Sn may exhibit topological properties.^29,57,58^ Some DFT studies have considered slab models of biaxially strained α-Sn. DFT simulations of strained α-Sn on InSb have been conducted with a small number of layers of both materials.^29,59^ The DFT+U approach has reproduced the effects of strain and compared well with experimental data.^31,57,59^

Here, we perform first-principles calculations using DFT+U(BO) for a (110) trilayer semiconductor/tunnel barrier/metal interface composed of the materials InSb/CdTe/α-Sn, owing to their relevance to current Majorana search experiments.^12,28^ To date, DFT studies of large interface slab models with a vacuum region have not been conducted for these interfaces. Previously, the results of DFT+U(BO) for InSb(110) have been shown to be in good agreement with angle-resolved photoemission spectroscopy (ARPES) experiments.^60^ Here, we also compare the results of DFT+U(BO) to ARPES for α-Sn (Section α-Sn) and CdTe (Section CdTe). Excellent agreement with experiment is obtained. In particular, for CdTe the *z*-unfolding scheme (Section *z*-Unfolding) helps resolve the contributions of different *k*_*z*_ values and modeling the 2 × 2 surface reconstruction reproduces the spectral signatures of surface states. We then proceed to study the bilayer interfaces of InSb/CdTe, CdTe/α-Sn, and InSb/α-Sn (Section Bilayer Interfaces). Finally, to assess the effectiveness of the tunnel barrier, we study trilayer interfaces with 2 to 16 monolayers (0.5 to 3.5 nm) of CdTe inserted between the InSb substrate and the α-Sn (Section Trilayer Interfaces). This thickness is within the thickness range of CdTe shells grown on InSb nanowires. For all interfaces, our simulations provide information on the band alignment and the presence of MIGS. We find that 16 layers of CdTe (about 3.5 nm) form an effective tunnel barrier, insulating the InSb from the α-Sn. However, this may be detrimental for transport at the interface. Based on this, we estimate that the relevant thickness regime for tuning the coupling between InSb and Sn may be in the range of 6–10 layers of CdTe.

## Methods

### *z*-Unfolding

**Figure 1:**
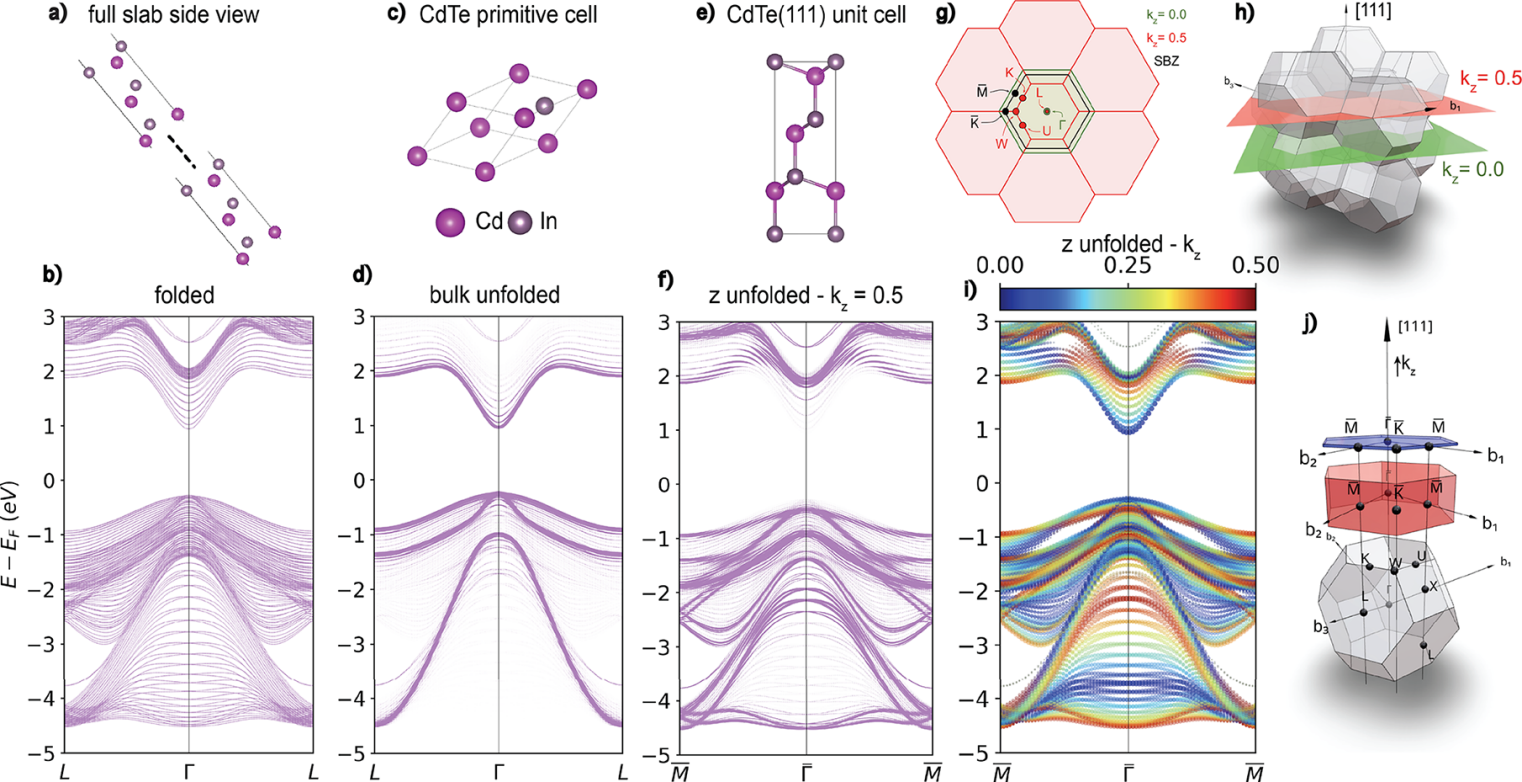
(a) Side view of the CdTe(111) slab. (b) Folded band structure of CdTe(111) 25 monolayer slab. (c) Primitive unit cell of CdTe (d) bulk-unfolded band structure. (e) Unit cell of CdTe(111) slab used in *z*-unfolding. (f) *z*-Unfolded band structure along the *k*-path *M̅*–Γ̅–*M̅* for *k*_*z*_ = 0.5. (g) Intersecting planes slice through the bulk BZ for *k*_*z*_ = 0 (green) and *k*_*z*_ = 0.5 (red) with the SBZ indicated. (h) Tessellated bulk BZs showing (111) orientated intersecting planes for given *k*_*z*_ values. (i) *z*-Unfolded band structure along the *k*-path *M̅*–Γ̅–*M̅* as a function of *k*_*z*_. (j) FCC bulk BZ (gray), (111) unit-cell BZ (red), and (111) surface BZ (blue).

Simulations of large supercell models produce complex band structures with a large number of bands, as shown in Figure 1a,b for a CdTe(111) slab with 25 atomic layers, whose band structure was calculated using PBE+U(BO), as described in the Supporting Information. Band structure unfolding is a method of projecting the band structure of a supercell model onto the appropriate smaller cell.^60,61^ This can help resolve the contributions of states emerging from *e.g.*, defects and surface reconstructions vs the bulk bands of the material. In addition, it can facilitate the comparison to angle-resolved photoemission spectroscopy (ARPES) experiments. The “bulk band unfolding” scheme^60^ projects the supercell band structure onto the primitive unit cell, illustrated in Figure 1c. The resulting band structure, shown in Figure 1d, appears bulk-like. Bulk-unfolded band structures have been shown to compare well with ARPES experiments using high photon energies, which are not surface sensitive owing to the large penetration depth.

The “*z*-unfolding” scheme^60^ projects the band structure of a slab model with a finite thickness onto the Brillouin zone (BZ) of a single layer of the slab supercell with the same orientation, illustrated in Figure 1e. The resulting band structure, shown in Figure 1f, contains extra bands that are not present in the bulk-unfolded band structure. The extra bands originate from different *k*_*z*_ values in the 3D primitive Brillouin zone projecting onto the surface Brillouin zone (SBZ), creating overlapping paths. For example, panel Figure 1g shows cross sections through the BZ at values of *k*_*z*_ = 0 and *k*_*z*_ = 0.5. The bulk-paths of Γ–*L*, Γ–*K*, and Γ–*X* all overlap with the surface k-path Γ̅–*M̅*, possibly with contributions from additional paths, such as *X*–*U*. The plane cuts at different *k*_*z*_ values are derived from the tessellated bulk BZ structure, shown in Figure 1h. When *z*-unfolding is performed, the value of *k*_*z*_ may be treated as a free parameter. The dependence on *k*_*z*_ manifests as a smooth change in the spectral function over the possible range of *k*_*z*_ which varies the mixture of different constituent bulk-paths that overlap the SBZ-path, as shown in Figure 1i for Γ̅–*M̅*. The BZ for *z*-unfolding is a surface BZ with a finite thickness, shown in red in Figure 1j. The simulation cell for the DFT calculations is set up to be the corresponding real-space unit cell. The *z*-unfolded k-paths are parallel to the (111) surface at a constant value of *k*_*z*_.

In ARPES experiments, the relation of the experimental spectra to *k*_*z*_ might not be straightforward. First, the dependence of the inelastic mean free path of the electrons on their kinetic energy is given by the universal curve.^62,63^ Using photon energies that correspond to a small mean free path is advantageous for probing surface states. However, it can produce prominent *k*_*z*_ broadening due to the Heisenberg uncertainty principle^64−67^ that implies integration of the ARPES signal over *k*_*z*_ through the broadening interval. Second, deviations of the photoemission final states from the free electron approximation can cause contributions from different values of *k*_*z*_ to appear in the ARPES spectra. The photoelectrons are often treated as free electrons, based on the assumption that the photoelectron kinetic energy is much larger than the modulations of the crystal potential. In this case, *k*_*z*_ for a given photoelectron kinetic energy, *E*_*k*_, and the in-plane momentum, **K**_**∥**_, is one single value, which is determined by
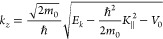
1where *m*_0_ is the free-electron mass and *V*_0_ the inner potential in the crystal. However, a considerable body of evidence has accumulated that the final states, even in metals,^68,69^ and to a greater extent in complex materials such as transition metal dichalcogenides,^70,71^ can significantly deviate from the free electron approximation. Such deviations can appear, first, as nonparabolic dispersions of the final states and, second, as their multiband composition. The latter means that for given *E*_*k*_ and **K**_**∥**_ the final-state wave function Φ_*f*_ incorporates a few Bloch waves ϕ_*k*_*z*__ with different *k*_*z*_ values, Φ_*f*_ = ∑_*k*_*z*__*A*_*k*_*z*__ϕ_*k*_*z*__, which give comparable contributions to the total photocurrent determined by the *A*_*k*_*z*__ amplitudes.^68^ A detailed theoretical description of the multiband final states, treated as the time-reversed low-energy electron diffraction (LEED) states^65^ within the wave function matching approach, as well as further examples for various materials can be found in refs (70) and (71) and the references therein. An insightful analysis of the multiband final states extending into the soft-X-ray photon energies can be found in ref (69). A rigorous analysis of final state effects in ARPES is beyond the scope of this work. Here, we will only mention that all these effects trace back to hybridization of free-electron plane waves through the higher Fourier components of the crystal potential. In cases where significant *k*_*z*_ broadening and/or final states effects are present, *z*-unfolding, rather than bulk unfolding, should be used in order to resolve the contributions of different *k*_*z*_ values to the measured spectrum. This is demonstrated for CdTe in Section CdTe, where the final states appear to incorporate two Bloch waves with *k*_*z*_ = 0 and *k*_*z*_ = 0.5.

### Interface Model Construction

DFT calculations were conducted with the Vienna Ab Initio Simulation Package (VASP)^72^ using the Perdew, Burke, and Ernzerhof (PBE)^73,74^ functional with Hubbard *U* corrections,^75^ whose values were machine learned using Bayesian optimization (BO).^52^ Additional computational details are provided in the Supporting Information. All interface models were constructed using the experimental InSb lattice constant value of 6.479 Å,^76^ assuming that the epitaxial films of CdTe and α-Sn would conform to the substrate. The length of two monolayers of a (110) slab was 4.5815 Å in the *z*-direction. A vacuum region of around 40 Å was added to each slab model in the *z*-direction to avoid spurious interactions between periodic replicas. The surfaces of all slab models were passivated by pseudohydrogen atoms such that there were no surface states from dangling bonds.^77^ Despite α-Sn being a semimetal, passivation is required to remove spurious surface states, as shown in the Supporting Information. The InSb/CdTe interface structure has In–Te and Sb–Cd bonds with each In interface atom connected to 3 Sb and 1 Te atoms. The configuration with In–Cd and Sb–Te bonds was also considered and was found to be less stable by 1.33 eV. Ideal interfaces were considered with no intermixing, and no relaxation of the interface atoms was performed.

When constructing such slab models, it is necessary to converge the number of layers to avoid quantum size effects and approach the bulk properties.^56^ For InSb, it has previously been shown that 42 monolayers are sufficiently converged.^60^ Plots of the band gap vs the number of atomic layers for CdTe(110) and α-Sn (110) slabs are provided in the Supporting Information. CdTe was deemed converged with 42 monolayers with a gap value of 1.23 eV, which is only slightly larger than the bulk PBE+U(BO) value. The *z*-unfolded band structures of CdTe(111) were calculated for a 40 monolayer slab. A 26 monolayer slab model was used to simulate the 2 × 2 reconstruction, due to the higher computational cost of the 2 × 2 supercell. Structural relaxation was performed for the top two monolayers of the 2 × 2 reconstruction. For the slab of (110) α-Sn, 70 monolayers were needed to close the gap at the zero-gap point of the semimetal, which corresponds to around 16 nm. The trilayer slab models comprised 42 layers of InSb, 70 layers of α-Sn, and between 0 and 16 layers of CdTe in two-layer increments, amounting to a total slab thickness of around 300 nm (not including vacuum). The (110) bilayer slab models comprised 42 layers of CdTe and InSb, and 70 layers of α-Sn as these were deemed converged.

## Results and Discussion

### α-Sn

**Figure 2:**
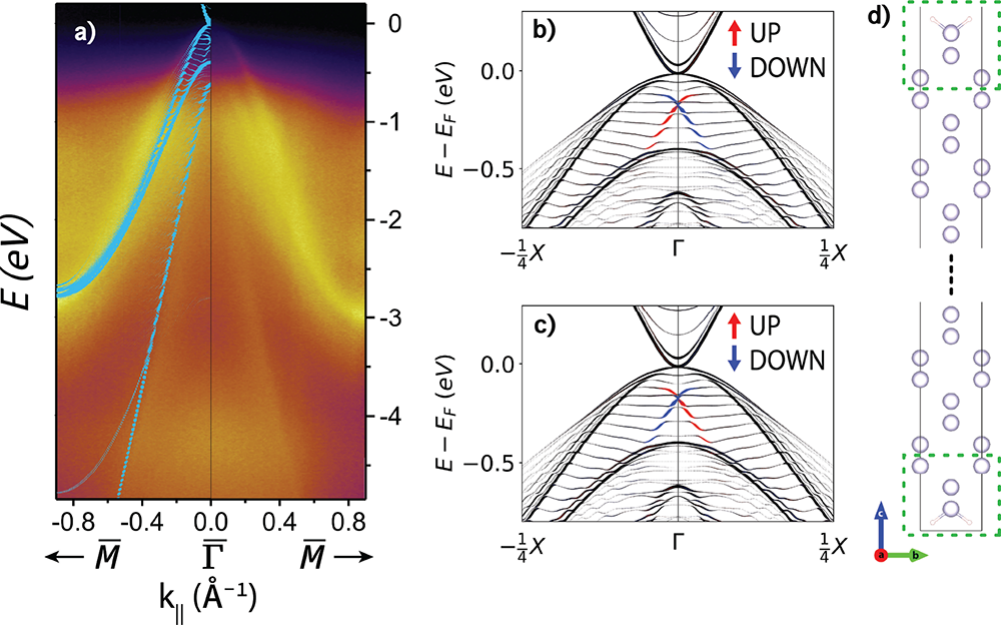
Electronic structure of α-Sn: (a) Bulk-unfolded band structure of an α-Sn (001) slab with 51 atomic layers (light blue) compared with ARPES data for a sample of the same thickness. The point *M̅* is at 0.9298 Å^–1^. The ARPES data is cutoff at 0.9 Å^–1^ due to experimental artifacts at the edges. Spin-polarized band structures projected onto (b) the top surface atoms and (c) the bottom surface atoms, indicated by the green boxes on the slab structure illustrated in panel d.

ARPES experiments were conducted for a 51 monolayer thick α-Sn(001) film, using a photon energy of 63 eV, as described in the Supporting Information. Figure 2a shows the bulk unfolded PBE+U(BO) band structure for a 51 monolayer thick α-Sn(001) slab, compared to the ARPES data. The point *M̅* is at 0.9298 Å^–1^. The ARPES data is cutoff at 0.9 Å^–1^ due to experimental artifacts at the edges. The PBE+U (BO) band structure is in excellent agreement with ARPES. The top of the valence band in the ARPES and the simulated band structure line up, and the bulk bands are reproduced well. The bandwidth of the heavy hole band, Γ_8_, is slightly underestimated, consistent with ref (60). This is corrected by the HSE functional, as shown in the Supporting Information for a bulk unit cell of α-Sn with a (001) orientation. However, it is not feasible to use HSE for the large interface models studied here, owing to its high computational cost.

The previously reported topological properties of α-Sn slabs are also observed here.^30−34,38,39,59^ The spin-polarized topological surface state (TSS) is shown in panels b and c of Figure 2 for a (001) 51 monolayer slab along the *X*–Γ–*Xk*-path. As expected, the TSS is characterized by a linear dispersion with the top and bottom surfaces having opposite spin polarization. The associated Rashba-like surface states are also observed along the *K*–Γ–*Kk*-path, as shown in the Supporting Information. This linear surface state is also observed in the (110) slabs used to construct the bilayer and trilayer models. Notably there is an energy gap between the top and bottom TSSs, which closes at 70 layers, the same thickness at which the band gap closes. This gap is possibly induced by the hybridization of the top and bottom surface states in under-converged slabs. We note that the effect of strain on the electronic structure of α-Sn is not studied here.

### CdTe

**Figure 3:**
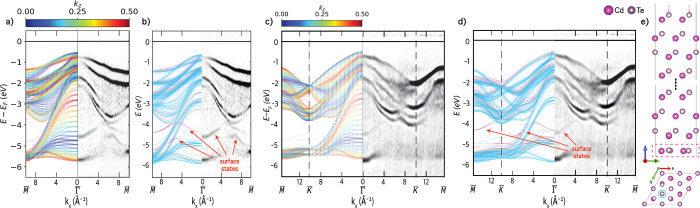
Electronic structure of CdTe: *z*-Unfolded band structures of CdTe(111) compared with second-derivative map of ARPES data (black and white). Adapted with permission from ref (78). Copyright 2015 American Physical Society. *z*-Unfolded band structures compared to ARPES data along (a, b) Γ̅–*M̅* and (c, d) Γ̅–*K̅*–*M̅*. (a, c) Dependence of the band structure on *k*_*z*_. (b, d) Mixture of *k*_*z*_ = 0.0 and *k*_*z*_ = 0.5 (cyan) for a model with a 2 × 2 surface reconstruction with the contributions of the surface atoms shown in pink. The DFT band structures are shifted by −0.25 eV and stretched by a factor of 1.22 for comparison. (e) Illustration of the 2 × 2 surface reconstruction with the Cd atom removed indicated by a blue circle. The atoms used for the surface projection are indicated by a pink dashed box.

Figure 3 shows a comparison of band structures obtained using PBE+U(BO) to the ARPES experiments of Ren et al.^78^ for CdTe(111). Ren et al. collected ARPES data at photon energies of 19, 25, and 30 eV. Here, we compare our results with the second-derivative maps of the ARPES data taken at 25 eV along the *k*-paths Γ̅–*M̅* (panels a and b) and Γ̅–*K̅*–*M̅* (panels c and d). The original data has been converted to gray scale. To facilitate the qualitative comparison of the DFT band structure features with the ARPES experiment, we apply a Fermi energy shift of 0.25 eV to line up the valence band maximum (VBM) and a stretch factor of 1.22 to compensate for the bandwidth underestimation of PBE+U(BO), particularly for bands deep below the Fermi energy.^79^ Bandwidth underestimation by PBE+U(BO) compared with HSE and ARPES has also been reported for InAs and InSb in.^60,80^ The original computed band structure without the shift and stretch is provided in the Supporting Information.

Owing to the low mean free path at this photon energy, the spectrum appears integrated over a certain *k*_*z*_ interval and surface contributions are readily visible in the ARPES.^62,63^ To account for the different *k*_*z*_ contributions, the *z*-unfolding method was employed, as described above. Panels (a) and (c) show the *z*-unfolded band structures as a function of *k*_*z*_ for slab models without a surface reconstruction (figures with single values of *k*_*z*_ are provided in the Supporting Information). This is used determine which *k*_*z*_ values are likely present in the experiment. A mixture of *k*_*z*_ = 0 and *k* = 0.5 provides the best agreement with the ARPES data. This combination of *k*_*z*_ values is used for the DFT data shown in cyan in panels (b) and (d). This is consistent with the *k*_*z*_ broadening with contributions centered around *k*_*z*_ = 0 and *k* = 0.5 often present in ARPES data taken at low mean field path energies in gapped materials.^64,65,81^

To account for the presence of surface states, we modeled the CdTe(111)A-(2 × 2) surface reconstruction,^82^ illustrated in panel (e). A comparison to the unreconstructed surface is provided in the Supporting Information. The atom-projected band structures of the bottom layer (indicated by pink dashed box) are plotted in pink in panels b and d. The additional bands arising from the surface reconstruction are in close agreement with the bands in the ARPES labeled as surface states by Ren et al., indicated by red arrows. These surface states are unaffected by the choice of *k*_*z*_. By accounting for the contributions of different *k*_*z*_ values and for the presence of surface states, excellent agreement with experiment is achieved, with the DFT band structures reproducing all the features of the ARPES.

### Bilayer Interfaces

**Figure 4:**
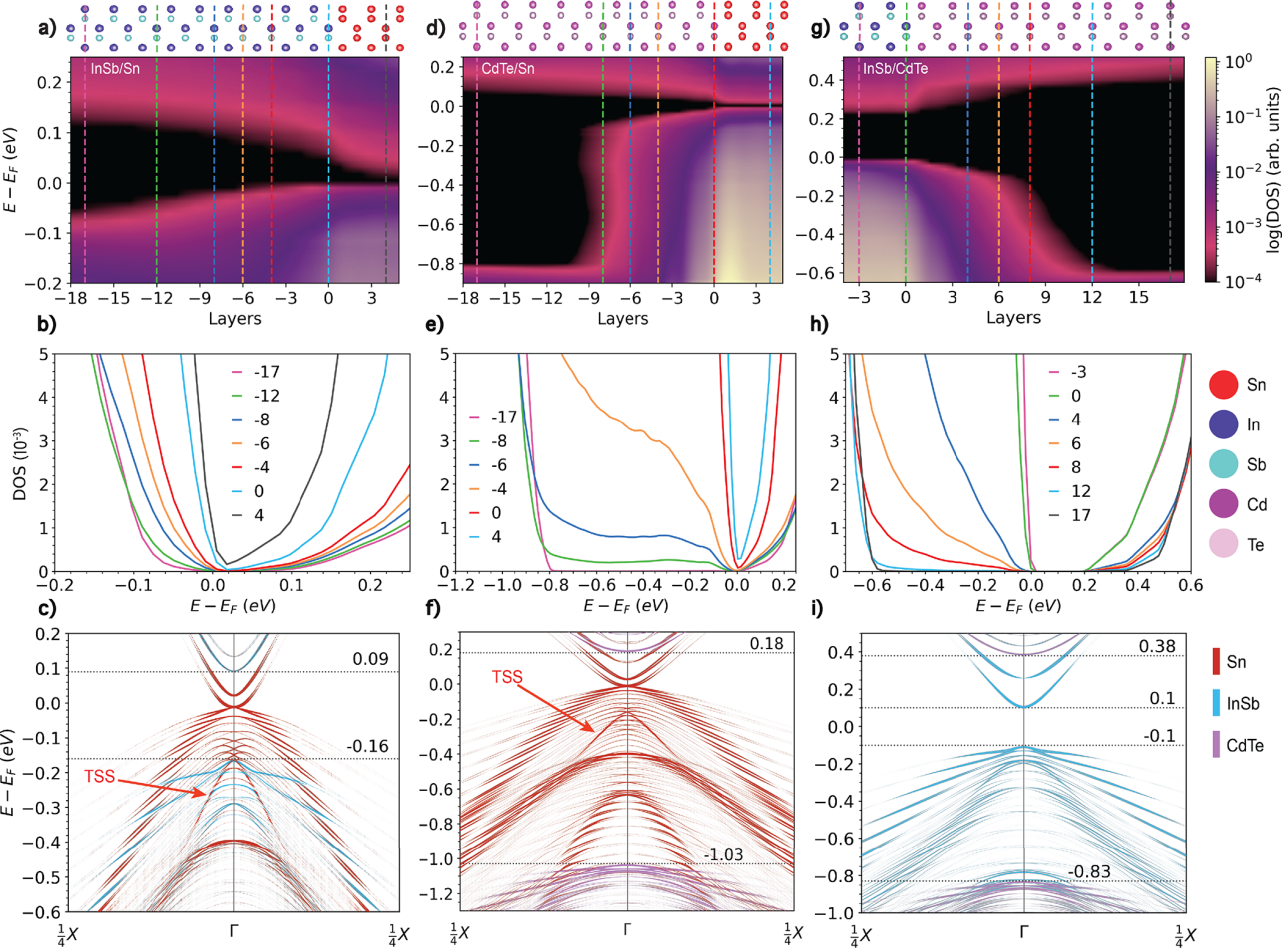
Electronic structure of bilayer interfaces: Density of states in the (a) InSb/α-Sn, (d) CdTe/α-Sn, and (g) InSb/CdTe interfaces as a function of position. The atomic layers are numbered based on distance from the interface, which is located at zero. The structure of each interface is illustrated on top. Local density of states for selected layers in the (b) InSb/α-Sn, (e) CdTe/α-Sn, and (h) InSb/CdTe interfaces, indicated by dashed lines in the same colors in panels a, d, and g, respectively. Element projected band structures of the (c) InSb/α-Sn, (f) CdTe/α-Sn, and (i) InSb/CdTe interfaces, with bands originating from α-Sn colored in red, bands originating from InSb colored in light blue, and bands originating from CdTe colored in purple.

We begin by probing the local electronic structure at the InSb/α-Sn bilayer interface. Figure 4a shows the DOS as a function of position across the interface, indicated by the atomic layer number. Figure 4b shows the local DOS at select positions. The Fermi level is located at the semimetal point of the α-Sn and in the gap of the InSb. We note that the α-Sn appears as if it has a small gap due to an artifact of the 10^–4^ cutoff applied in the log plot in panels a and d. The local DOS plots shown in panels b and e and the band structure plots shown in panels c and f clearly show the semimetal point. No significant band bending is found for InSb, as expected from branching point theory.^83,84^ Based on the element-projected band structure, shown in panel c, the InSb conduction band minimum (CBM) lies 0.09 eV above the α-Sn semimetal point and the InSb VBM lies 0.16 eV below it. A linear TSS is present in the α-Sn. Based on an atom projected band structure, shown in the Supporting Information, the origin of this state is the top surface of α-Sn, adjacent to the vacuum region. A TSS is no longer present in the α-Sn layers at the interface with InSb, possibly owing to hybridization between the α-Sn and InSb.^59^ Metal-induced gap states (MIGS) are an inherent property of a metal/semiconductor interface, produced by the penetration of exponentially decaying metallic Bloch states into the gap of the semiconductor.^85−87^ The presence of MIGS manifests in Figure Figure 4a as a gradually decaying nonzero DOS in the band gap of the InSb in the vicinity of the interface. Figure 4b shows that the MIGS are prominent in the first few atomic layers and become negligible beyond 8 layers from the interface.

Figure 4d shows the DOS as a function of position across the CdTe/α-Sn interface, indicated by the atomic layer number. Figure 4e shows the local DOS at select positions. The Fermi level is located at the semimetal point of the α-Sn and in the gap of the CdTe. Based on the projected band structure, shown in panel f, the CdTe CBM is positioned 0.18 eV above the Fermi level and the CdTe VBM is located 1.03 eV below the Fermi level. This agrees with previous reports that interfacing with Sn brings the conduction band of the CdTe closer to the Fermi energy, with downward band-bending of 0.25 eV^88^ and 0.1 eV.^89^ We find a valence band offset of around 1 eV, similar to the (110) and (111) interfaces reported in the literature.^36,89−93^ Close to the interface there is a significant density of MIGS, which decay within about 10 layers (3–4 nm) into the CdTe. This suggests that this number of CdTe layers may be required for an effective tunnel barrier.

Figure 4g shows the DOS as a function of position across the InSb/CdTe interface, indicated by the atomic layer number. Figure 4h shows the local DOS at select positions. The band alignment is type-I with the CdTe band gap straddling the InSb band-edges. The Fermi level is close to the InSb VBM and around the middle of the gap of the CdTe. No band bending is found in either material. Based on the projected band structure, shown in panel i, the CdTe CBM lies 0.28 eV above the InSb CBM and the CdTe VBM lies 0.75 eV below the InSb VBM. These values are similar to the band offsets reported in refs (28), (94), and (95). Because the band gap of InSb is significantly smaller than that of CdTe, states from the InSb penetrate into the gap of the CdTe, similar to MIGS. These states decay gradually and vanish at a distance greater than 12 layers from the interface.

### Trilayer Interfaces

**Figure 5:**
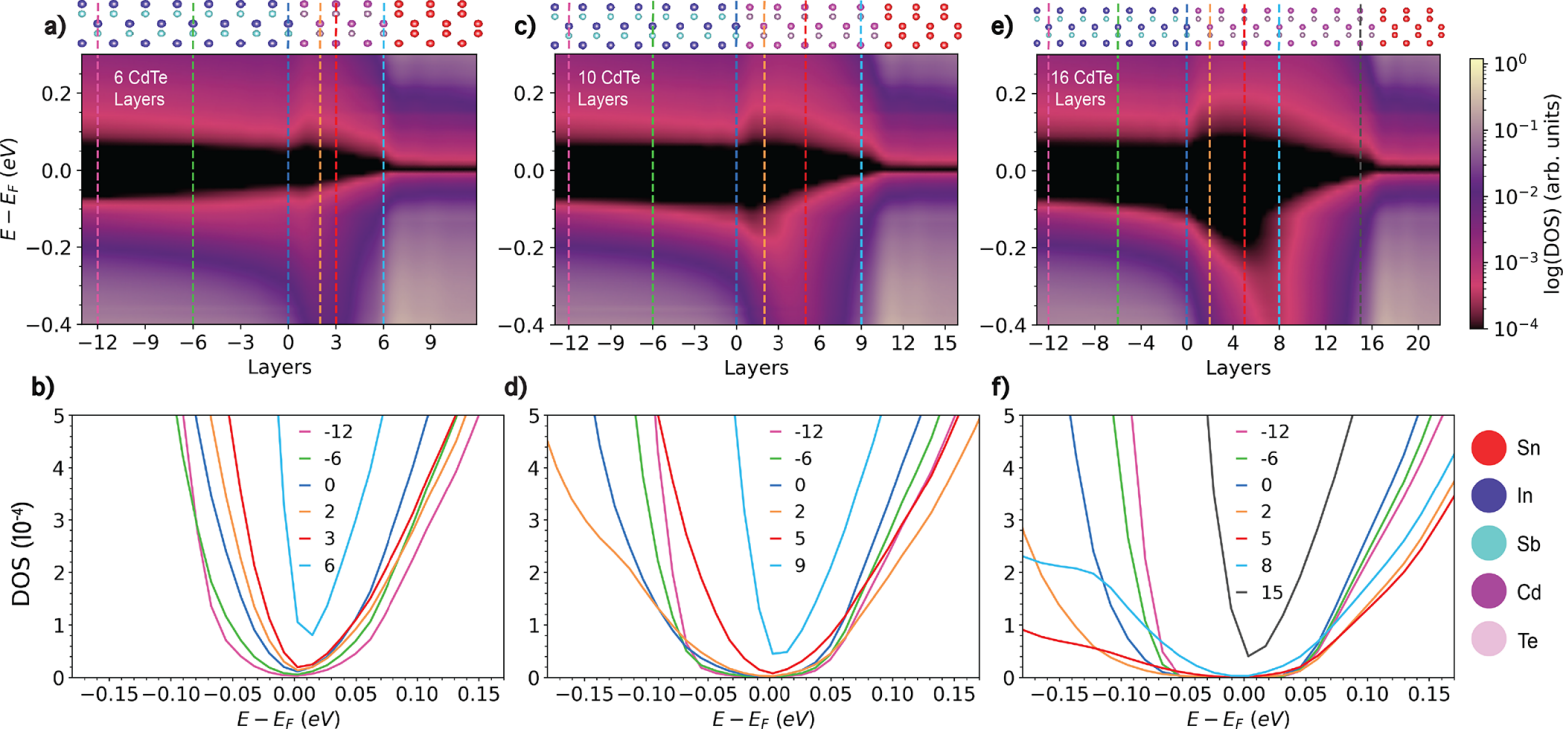
Electronic structure of InSb/CdTe/α-Sn trilayer interfaces: Density of states as a function of distance from the interface for (a) 6, (c) 10, and (e) 16 CdTe barrier layers. The atomic layers are numbered based on distance from the interface, which is located at zero. Interface structures are illustrated on top. (b, d, f) Local density of states for selected layers, indicated by dashed lines in the same colors in panels a, c, and e, respectively.

Figure 5 shows the DOS as a function of position across InSb/CdTe/α-Sn trilayer interfaces with varying thickness of the CdTe tunnel barrier. Interfaces with 6, 10, and 16 layers of CdTe are shown here, and additional results for interfaces with 2, 4, and 8 layers are provided in the Supporting Information. The position across the interface is indicated by the atomic layer number, with the layer of InSb closest to the CdTe considered as zero. Panels a and b show that with 6 atomic layers of CdTe, the MIGS from the α-Sn penetrate through the tunnel barrier into the first 12 layers of the InSb. For a thin layer of CdTe, the band gap is expected to be significantly larger than the bulk value because of the quantum size effect (see the gap convergence plot in the Supporting Information). However, owing to the presence of MIGS, the gap of the CdTe remains considerably smaller than its bulk value. With 10 layers of CdTe, shown in panels c and d, there is still a significant presence of MIGS throughout the CdTe, which decay by 6 layers into the InSb. Panels e and f show that with 16 layers of CdTe, the InSb is completely insulated from MIGS coming from the α-Sn. The gap of the CdTe reaches a maximum of around 0.3 eV at a distance of 5 layers from the InSb. This is because MIGS from the α-Sn penetrate into the CdTe from one side, whereas states from the InSb penetrate from the other side, such that the band gap of the CdTe never reaches its expected value.

**Figure 6:**
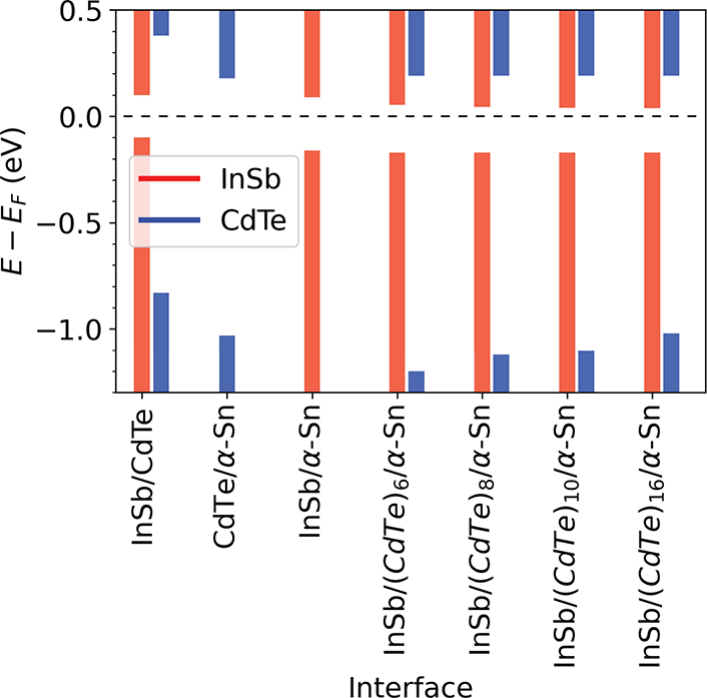
Valence and conduction band edge positions for InSb and CdTe in the bilayer and trilayer interfaces. The Fermi level is at the semimetal point of the α-Sn.

Figure 6 summarizes the band alignment at the bilayer and trilayer interfaces studied here. For the trilayer interfaces, the band alignment between the InSb and the α-Sn is not significantly affected by the presence of CdTe, as shown in the element-projected band structures in the Supporting Information. The α-Sn semimetal point remains pinned at the Fermi level, as in the bilayer InSb/α-Sn (see also Figure 4c). The InSb VBM remains at 0.17 eV below the Fermi level, similar to its position in the bilayer interface, regardless of the CdTe thickness. The InSb CBM position shifts slightly with the thickness of the CdTe from 0.09 eV above the Fermi level without CdTe, to 0.054 eV with 6 layers of CdTe, 0.04 eV with 10 layers, and 0.037 eV with 16 layers. This may be attributed to the quantum size effect, which causes a slight narrowing of the InSb gap because of the increase in the overall size of the system. Based on the element-projected band structures provided in the Supporting Information, the band edge positions of the CdTe are dominated by the interface with the α-Sn, rather than the interface with the InSb. The CdTe CBM remains at 0.18 eV above the Fermi level, as in the bilayer CdTe/α-Sn interface (see also Figure 4f), regardless of the number of layers. As the band gap of the CdTe narrows with increasing thickness, the CdTe VBM shifts from 1.24 eV below the Fermi level with 6 layers to 1.105 eV with 10 layers, and 1.05 eV with 16 layers, approaching the bilayer VBM position of 1.03 eV below the Fermi level with 42 layers. Although the band gap of the CdTe is significantly reduced due to MIGS, a type I band alignment with the InSb is maintained, similar to the bilayer InSb/CdTe interface (Figure 4g,i), as shown in Figure 5 panels a, c, and e.

**Figure 7:**
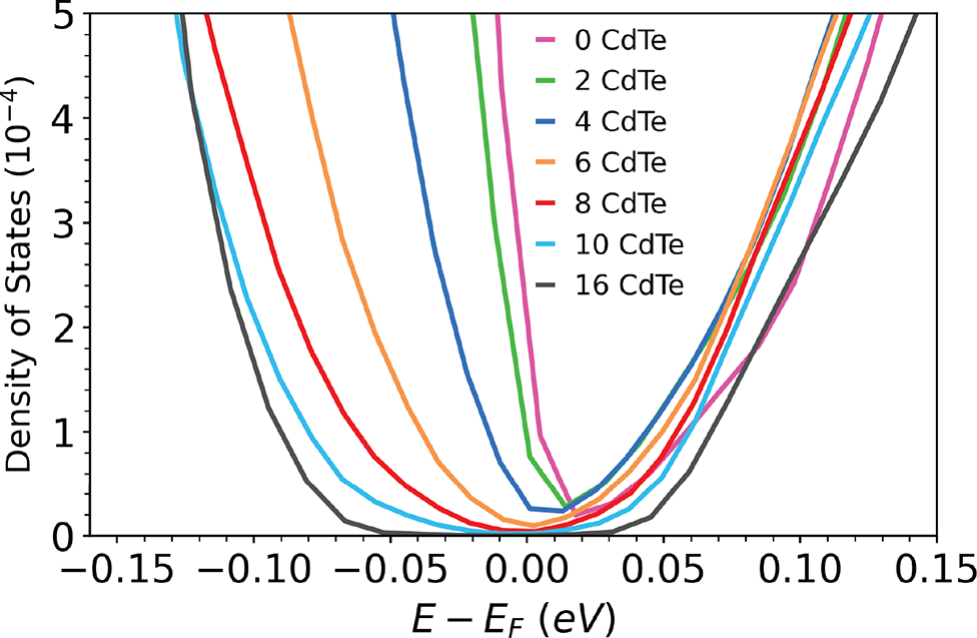
Density of states in the second InSb layer from the interface (layer −2 in Figure 5) as a function of the number of CdTe barrier layers.

Figure 7 shows the local DOS in the second layer of InSb from the interface as a function of the number of CdTe layers. Without CdTe and with two layers of CdTe, there is no band gap in the InSb close to the interface, owing to the significant density of MIGS. With 4 layers of CdTe, a gap starts to appear. With 6 layers of CdTe, the gap of the InSb close to the interface is still considerably narrower than its bulk value. The band gap in the second layer of InSb from the interface approaches its bulk value with 10 layers of CdTe and finally reaches it with 16 layers of CdTe. This suggests that 16 CdTe layers provide an effective barrier to electronically insulate the InSb from the α-Sn. With present-day methods, we are unable to calculate the current across the interface from first principles. It is reasonable to assume that a barrier of 16 layers or more (over 3.5 nm thick) would all but eliminate transport through the interface into the InSb. We surmise that the barrier thickness range where there is still some overlap between the wave functions of the α-Sn and the InSb, as indicated by the presence of MIGS, is the relevant regime to modulate the coupling strength between the two materials. This calls for experimental studies of the proximity effect at a semiconductor/superconductor interface with a tunnel barrier in the range of 6–10 layers, where MIGS still exist. We note, however, that the interface with β-Sn may have somewhat different characteristics in terms of the band alignment and the penetration depth of MIGS.

## Conclusion

In summary, we have used DFT with a Hubbard U correction machine-learned by Bayesian optimization to study CdTe as a prospective tunnel barrier at the InSb/α-Sn interface. The results of PBE+U(BO) were validated by comparing the band structures of slab models of α-Sn(001) and CdTe(111) with ARPES experiments (the PBE+U(BO) band structure of InSb(110) had been compared to ARPES experiments previously^60^). Excellent agreement with experiment is obtained for both materials. In particular, for the low-mean-free-path ARPES of CdTe, the *z*-unfolding scheme successfully reproduces the contributions of different *k*_*z*_ values, and modeling the 2 × 2 surface reconstruction successfully reproduces the contributions of surface states.

We then proceeded to use PBE+U(BO) to calculate the electronic structure of bilayer InSb/α-Sn, CdTe/α-Sn, and InSb/CdTe, as well as trilayer InSb/CdTe/α-Sn interfaces with varying thickness of CdTe. Simulations of these very large interface models were possible thanks to the balance between accuracy and computational cost provided by PBE+U(BO). We find that the most stable configuration of the InSb/CdTe interface is with In–Te and Sb–Cd bonding. MIGS penetrate from the α-Sn into the InSn and CdTe. Similarly, states from the band edges of InSb penetrate into the larger gap of the CdTe. No interface states are found in any of the interfaces studied here, in contrast to the EuS/InAs interface, for example, in which a quantum well interface state emerges.^96^

For all interfaces comprising α-Sn, the semimetal point is pinned at the Fermi level. For the trilayer interface, the band alignment between the InSb and the α-Sn remains the same as in the bilayer interface regardless of the thickness of the CdTe barrier, with the Fermi level closer to the conduction band edge of the InSb. The band edge positions of the CdTe are dominated by the interface with the α-Sn rather than the interface with InSb, with the conduction band edge being closer to the Fermi level. A type-I band alignment is maintained between CdTe and InSb with the gap of the former straddling the latter. The CBM of the CdTe is pinned whereas the VBM shifts upward toward the Fermi level as the gap narrows with the increase in thickness.

We find that 16 layers of CdTe (about 3.5 nm) serve as an effective barrier, preventing the penetration of MIGS from the α-Sn into the InSb. However, in the context of Majorana experiments, it is possible that a barrier thick enough to completely insulate the semiconductor from the superconductor would also all but eliminate transport. Therefore, we estimate that the relevant regime for tuning the coupling at the interface would be in the thickness range where some MIGS are still present. Thicker CdTe layers could be used to passivate exposed InSb surfaces. We note that the interface with the superconducting β-Sn, which is not lattice matched to InSb and CdTe, may have different characteristics than the interface with α-Sn. Careful experimentation is needed to establish a connection between proximity-induced superconductivity or other physical factors that affect the emergence of MZMs and the parameters of the interface, including the band alignment and the presence or absence of interface states and MIGS. Such experiments could be pursued, for example, by varying the CdTe barrier thickness within the range indicated by our simulations and measuring the induced superconducting gap in an InSb nanowire. This would help determine the optimal interface configuration for MZM devices.

We have thus demonstrated that DFT simulations can provide useful insight into the electronic properties of semiconductor/tunnel barrier/metal interfaces. This includes the interface bonding configuration, the band alignment, and the presence of MIGS (and, possibly, of interface states). Such simulations may be conducted for additional interfaces to explore other prospective material combinations. This may inform the choice of interface systems and the design of future Majorana experiments. More broadly, similar DFT simulations of interfaces may be performed to evaluate prospective tunnel barriers, *e.g.*, for semiconductor devices.

## Supporting Information Available

Computational details of DFT simulations; experimental details of ARPES of α-Sn; comparison of the HSE and PBE+U(BO) band structures of α-Sn and CdTe; effect of lattice parameter on the band structures of α-Sn and CdTe; topological properties of α-Sn; surface passivation of α-Sn; thickness convergence of α-Sn and CdTe; the 2 × 2 surface reconstruction of CdTe(111); additional results for the *z*-unfolded band structure of CdTe(111); contribution of the α-Sn surface to the band structure of bilayer InSb/α-Sn; band alignment of the trilayer InSb/CdTe/α-Sn interface; and additional results for the trilayer InSb/CdTe/α-Sn interface with 2, 4, and 8 layers of CdTe (PDF)
