# Correction to “First-Principles Assessment of CdTe as a Tunnel Barrier at the α-Sn/InSb Interface”

**DOI:** 10.1021/acsami.3c07924

**Published:** 2023-06-15

**Authors:** Malcolm J. A. Jardine, Derek Dardzinski, Maituo Yu, Amrita Purkayastha, An-Hsi Chen, Yu-Hao Chang, Aaron Engel, Vladimir N. Strocov, Moïra Hocevar, Chris Palmstrøm, Sergey M. Frolov, Noa Marom

**Affiliations:** † Department of Physics and Astronomy, University of Pittsburgh, Pittsburgh, Pennsylvania 15260, United States; ‡ Department of Materials Science and Engineering, Carnegie Mellon University, Pittsburgh, Pennsylvania 15213, United States; § Université Grenoble Alpes, CNRS, Grenoble INP, Institut Néel, 38000 Grenoble, France; ∥ Materials Department, University of California-Santa Barbara, Santa Barbara, California 93106, United States; ⊥ Paul Scherrer Institut, Swiss Light Source, CH-5232 Villigen PSI, Switzerland; # Department of Electrical and Computer Engineering, University of California-Santa Barbara, Santa Barbara, California 93106, United States; ∇ Department of Physics, Carnegie Mellon University, Pittsburgh, Pennsylvania 15213, United States; ○ Department of Chemistry, Carnegie Mellon University, Pittsburgh, Pennsylvania 15213, United States

The original version of this article (https://pubs.acs.org/doi/full/10.1021/acsami.3c00323) contains errors in the affiliations of An-Hsi Chen, Yu-Hao Chang, Aaron Engel, Vladimir N. Strocov, Moïra Hocevar, Chris Palmstrøm, and Noa Marom. These errors are misprints made by the publisher. They do not affect the scientific content of the paper. The correct affiliations are provided here:

An-Hsi Chen: Université Grenoble Alpes, CNRS, Grenoble INP, Institut Néel, 38000 Grenoble, France.

Yu-Hao Chang: Materials Department, University of California-Santa Barbara, Santa Barbara, California 93106, United States.

Aaron Engel: Materials Department, University of California-Santa Barbara, Santa Barbara, California 93106, United States.

Vladimir N. Strocov: Paul Scherrer Institut, Swiss Light Source, CH-5232 Villigen PSI, Switzerland.

Moïra Hocevar: Université Grenoble Alpes, CNRS, Grenoble INP, Institut Néel, 38000 Grenoble, France.

Chris Palmstrøm: Materials Department and Department of Electrical and Computer Engineering, University of California-Santa Barbara, Santa Barbara, California 93106, United States.

Noa Marom: Department of Materials Science and Engineering, Department of Physics, and Department of Chemistry, Carnegie Mellon University, Pittsburgh, Pennsylvania 15213, United States.

