# Supplementary material for: First-Principles Assessment of CdTe as a Tunnel Barrier at the α-Sn/InSb Interface
Source: ACS Appl Mater Interfaces. 2023 Mar 20;15(12):16288–98. doi: 10.1021/acsami.3c00323 (PMC10064317; doi:10.1021/acsami.3c00323)
Supplement: Supplementary file 1 — am3c00323_si_001.pdf [file am3c00323_si_001.pdf]

Supporting Information to: First Principles Assessment of CdTe as a Tunnel Barrier at the α-Sn/InSb Interface

Malcolm J. A. Jardine,[†,‡] Derek Dardzinski,[¶,‡] Maituo Yu,[¶] Amrita Purkayastha,[†] An-Hsi Chen,[§] Yu-Hao Chang,[‖] Aaron Engel,[‖] Vladimir N. Strocov,[⊥] Moïra Hocevar,[§] Chris Palmstrøm,[‖] Sergey M. Frolov,[†] and Noa Marom[*,¶,@,△]

†*Department of Physics and Astronomy, University of Pittsburgh, Pittsburgh, PA, 15260, USA*

‡*These authors contributed equally*

¶*Department of Materials Science and Engineering, Carnegie Mellon University, Pittsburgh, PA 15213, USA*

§*Univ. Grenoble Alpes, CNRS, Grenoble INP, Institut Néel, 38000 Grenoble, France*

‖*Materials Department, University of California-Santa Barbara, Santa Barbara, CA 93106, USA*

⊥*Paul Scherrer Institut, Swiss Light Source, CH-5232 Villigen PSI, Switzerland*

#*Department of Electrical and Computer Engineering, University of California-Santa Barbara, Santa Barbara, CA 93106, USA*

@*Department of Physics, Carnegie Mellon University, Pittsburgh, PA 15213, USA*

△*Department of Chemistry, Carnegie Mellon University, Pittsburgh, PA 15213, USA*

E-mail: nmarom@andrew.cmu.edu

Additional Methodological Details

Computational Details

DFT calculations were conducted using the Vienna Ab Initio Simulation Package (VASP)[1] with the projector augmented wave method (PAW).[2,3] The generalized gradient approximation (GGA) of Perdew, Burke, and Ernzerhof (PBE)[4] was employed to describe the exchange-correlation interactions among electrons with a Hubbard U correction.[5] The U values were machine learned using Bayesian optimization (BO).[6] Briefly, the BO objective function is formulated to reproduce as closely as possible the band structure obtained from the Heyd-Scuseria-Ernzerhof (HSE)[7] hybrid functional. The reference HSE calculations were conducted for bulk CdTe with a lattice parameter of 6.482 Å and α-Sn with a lattice parameter of 6.489 Å and compared to the results with the lattice constant of InSb, 6.479 Å, which was used for interface models. It was verified that using the lattice constant of InSb does not have an appreciable effect on the electronic properties of CdTe and α-Sn, as shown below.

The hyperparameters of our BO implementation are the coefficients α_1 and α_2, which assign different weights to the band gap vs. the band structure in the objective function, the number of valence and conduction bands used for the calculation of the objective function, N_b, and the parameter κ that controls the balance between exploration and exploitation in the upper confidence bound acquisition function. For InSb the values of $U_{eff}^{In,p} = -0.2$ and $U_{eff}^{Sb,p} = -6.1$ were used, following Ref.[6,8] It has been shown that PBE+U(BO) produces a band structure in good agreement with ARPES for InSb .[8]

Because α-Sn is a semi-metal, only the band shape was considered in the optimization, *i.e.* α_1 was set to 0 and $\alpha_2 = 1$.[9]The other BO hyperparameters used for Sn were $\kappa = 7.5$ and $N_b = (5, 5)$. This resulted in a value of $U_{eff}^{Sn,p} = -3.04$ eV, slightly different than in Refs.,[10–12] which used empirical methods to choose a U value that yields a correct band ordering. As shown in Ref.,[9] PBE+U(BO) reproduces the correct band ordering of α-Sn

with the band inversion at the Γ point, in agreement with other studies using DFT+U.[13,14]

For CdTe, we applied a U correction to both the Cd-d orbitals and Te-p orbitals, unlike earlier studies.[15,16] The hyperparameters used for CdTe were $\kappa = 7.5$, $N_b = (5, 5)$, $\alpha_1 = 0.5$ and $\alpha_2 = 0.5$. The latter two parameters were chosen to assign equal weights to the band gap and the band shape. This led to U values of $U_{eff}^{Cd,d} = 7.381$ and $U_{eff}^{Te,p} = -7.912$. The Cd-d U value obtained here is similar to the 7 eV used in Ref.[16] and somewhat lower than $U_{eff}^{Cd,d} = 8.3$ eV in Ref.[15] The gap of 1.21 eV, obtained here by applying the Hubbard U correction to both the Te-p states and the Cd-d states is closer to experimental values of around 1.5 eV[17,18] and the HSE value of 1.31 eV than previous calculations.[15]

Spin-orbit coupling (SOC) was used in all calculations and dipole corrections were applied to slab models.[19] The tags used for convergence of calculations were BMIX = 3, AMIN = 0.01, ALGO = Fast, and EDIFF = $1{\cdot}10^{-5}$. The kinetic energy cutoff was set to 400 eV for all bulk calculations and 350 eV for surface and interface slab models. A $9 \times 9 \times 9$ k-point mesh was used for bulk calculations and a k-point mesh of $7 \times 7 \times 1$ was used for surface and interface calculations. All interface density of states (DOS) calculations used a k-point mesh of $13 \times 13 \times 1$.

The pseudo-hydrogen fractional charges utilized to passivate each atom were 1.25 for In and 0.75 for Sb in InSb, 1.5 for Cd and 0.5 for Te in CdTe, and 1 for Sn. Structural relaxation of the pseudo-hydrogen atoms was performed until the maximal force was below 0.001 eV/Å.

All band structure and density of states plots were generated using the open-source Python package, VaspVis,[9] which is freely available from The Python Package Index (PyPI) via the command: *pip install vaspvis*, or on GitHub at: `https://github.com/DerekDardzinski/vaspvis`

ARPES Experimental details

The α-Sn samples were grown by molecular beam epitaxy on an In-terminated c(8 $\times$ 2) InSb(001) surface prepared by atomic hydrogen cleaning. 51 monolayers (16.5 nm) of α-Sn were deposited as calibrated via Rutherford backscattering spectrometry. Growth was performed at a substrate temperature of -20 °C and a base pressure better than $1{\cdot}10^{-10}$ Torr. The ARPES measurements were taken at Beamline 10.0.1.2 at the Advanced Light Source in Berkeley. The base pressure was better than $5{\cdot}10^{-11}$ Torr while the sample temperature was held at 68 K. The sample was illuminated with 63 eV p-polarized light and spectra were collected using a Scienta R4000 detector with energy resolution better than 40 meV and angular resolution better than 0.1°. The sample was transferred via vacuum suitcase with a base pressure better than $\cdot 10^{-11}$ Torr between the growth chamber and beamline. A photon energy of 63 eV corresponds to a $\mathbf{k}_z$ approximately 0.15 Å^{-1} above the $\mathbf{\Gamma}_{002}$ point.

Additional Results

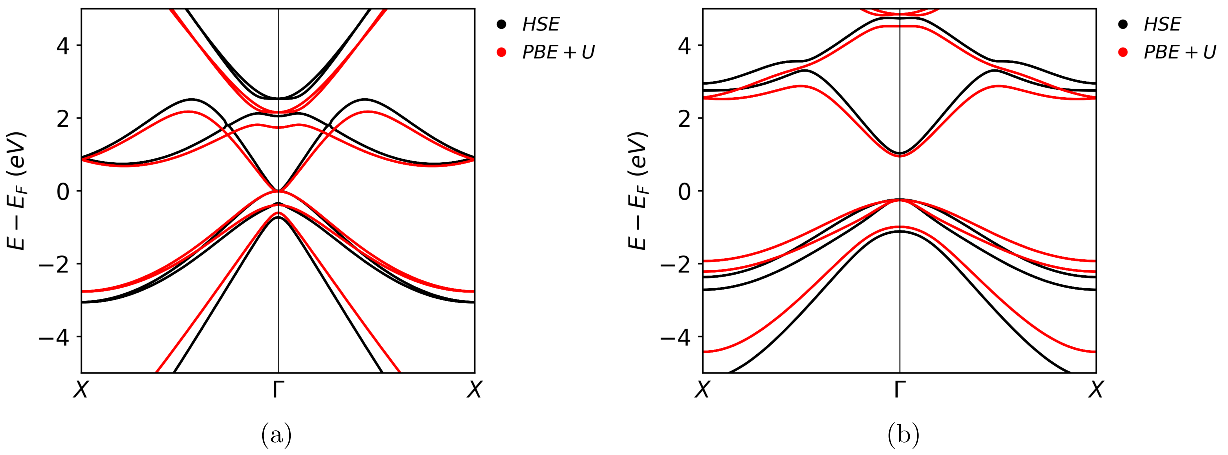

Figure S1: Comparison of the HSE and PBE+U(BO) band structures of (a) α-Sn and (b) CdTe. The band width is slightly underestimated by PBE+U(BO). This is the reason for the stretch factor applied in the comparison to ARPES for CdTe.

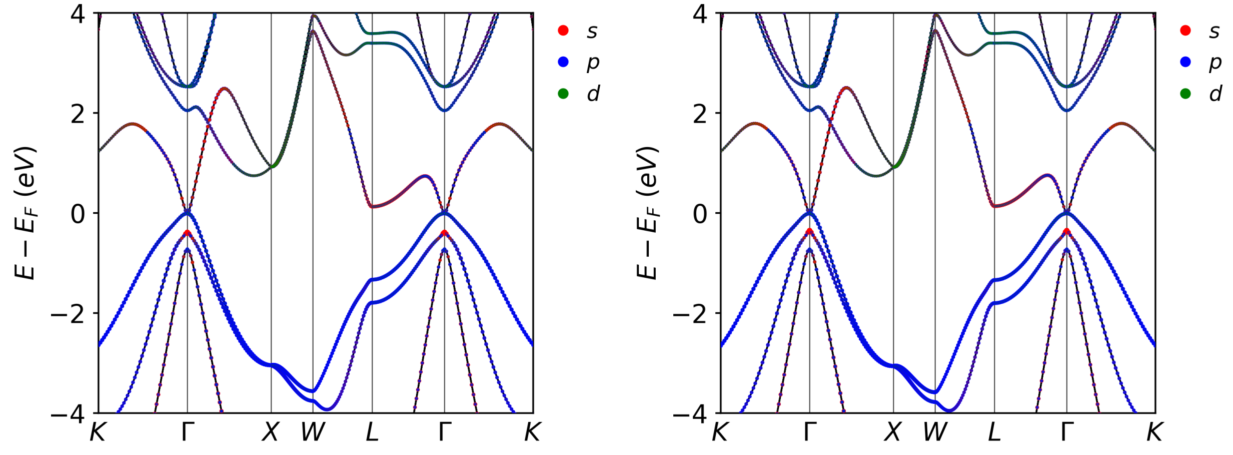

(a) (b)

Figure S2: Comparison of the HSE band structures of α-Sn obtained with the lattice constant of (a) 6.489 Å of α-Sn and (b) 6.479 Å of InSb. The small difference in the lattice constant has no appreciable effect on the electronic properties.

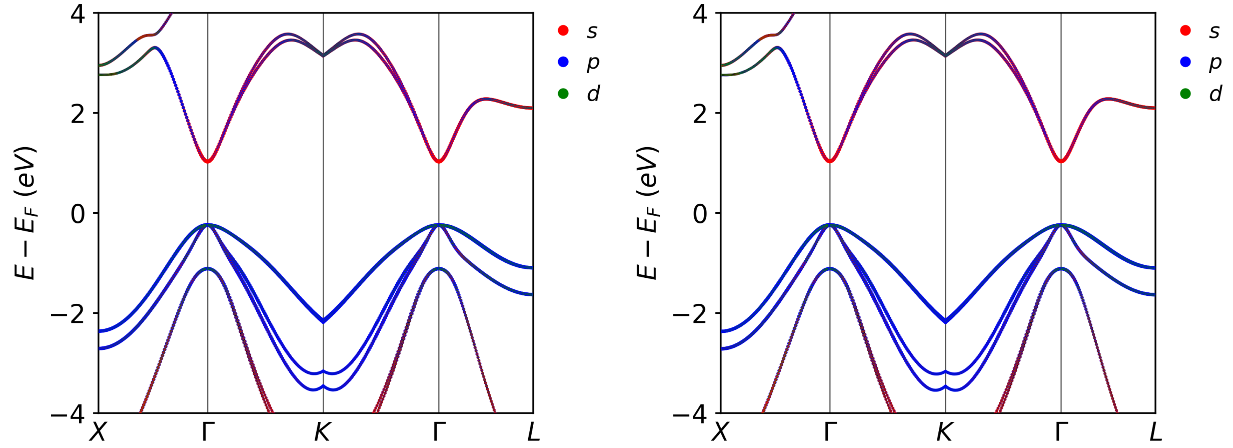

(a) (b)

Figure S3: Comparison of the HSE band structures of CdTe obtained with the lattice constant of (a) 6.482 Å of CdTe and (b) 6.479 Å of InSb. The band gaps are 1.266718 eV and 1.270622 eV respectively. The small difference in the lattice constant has no appreciable effect on the electronic properties.

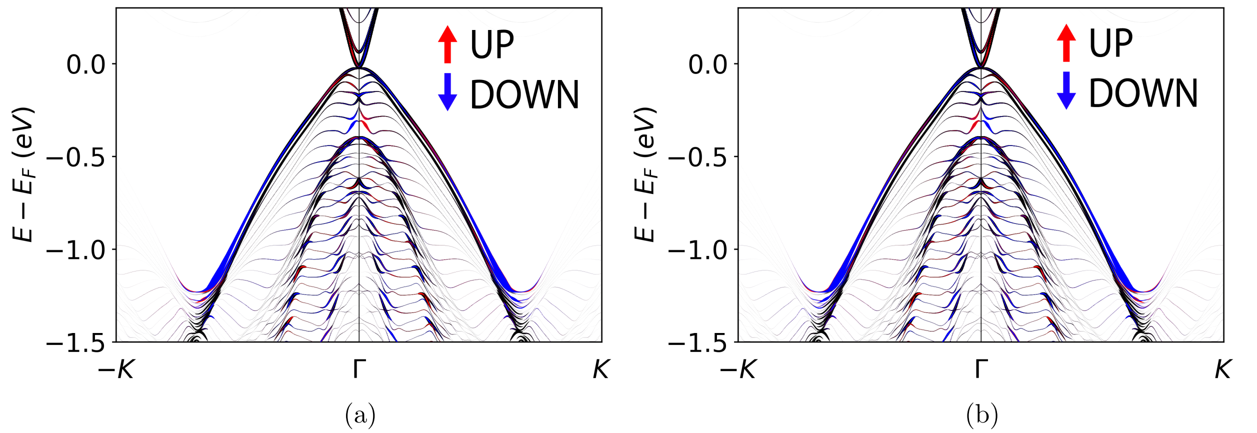

Figure S4: Topological properties of α-Sn: Band structure along the $K - \Gamma - K$ k-path obtained with PBE+U(BO) for an unstrained α-Sn(001) slab with 51 atomic layers. The spin decomposed output has been projected onto the (a) top and (b) bottom two monolayars of the slab, highlighting the TSS1 state and the RSS state on the surface of the slab. The RSS up and down spin polarized surface states are mostly degenerate.

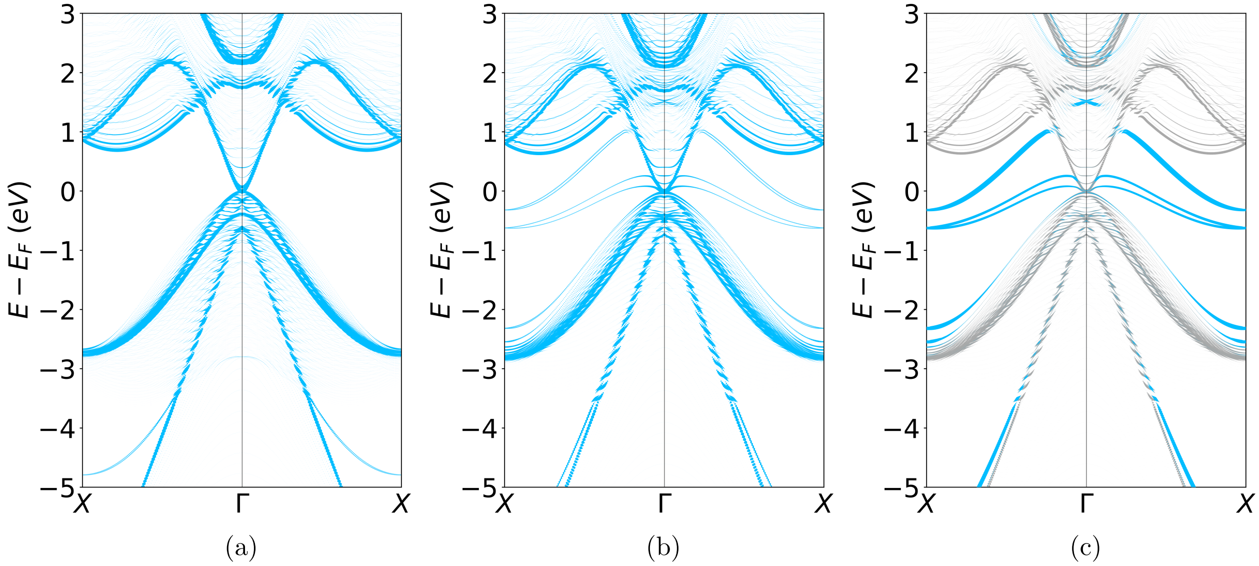

Figure S5: Surface passivation of α-Sn: Band structures of an α-Sn slab with 41 monolayers (a) with surface passivation and (b) without surface passivation, showing the spurious states due to dangling bonds. (c) Projected band structure with the contributions of the top two monolayers of a slab with no passivation colored in blue. This shows that the additional states surface states, which disappear when the dangling bonds are passivated.

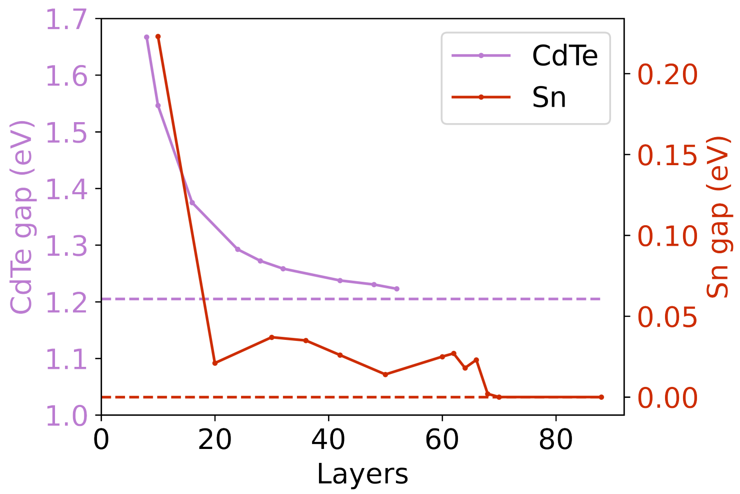

Figure S6: Thickness convergence of α-Sn and CdTe: The Γ-point band gap of CdTe(110) and α-Sn(110) as a function of number of atomic layers. Dashed lines denote the PBE+U(BO) bulk limit of 1.205 eV for CdTe and 0 eV (zero-gap) for Sn. Oscillatory behavior is observed for α-Sn(110). Similar behavior has been reported in other materials with a TSS.[20] This behavior could arise from hybridization of the top and bottom surface states, which may cause finite size effects such as band gap oscillations with layer number in topological crystalline insulators.[21,22]

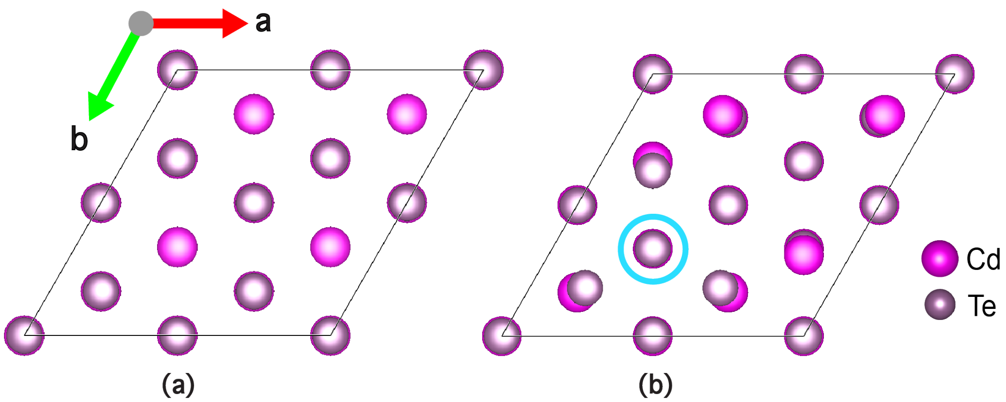

Figure S7: The 2×2 surface reconstruction of CdTe(111): Top view of (a) un-reconstructed surface and (b) reconstructed and relaxed surface with the removed Cd atom indicated by a blue circle.

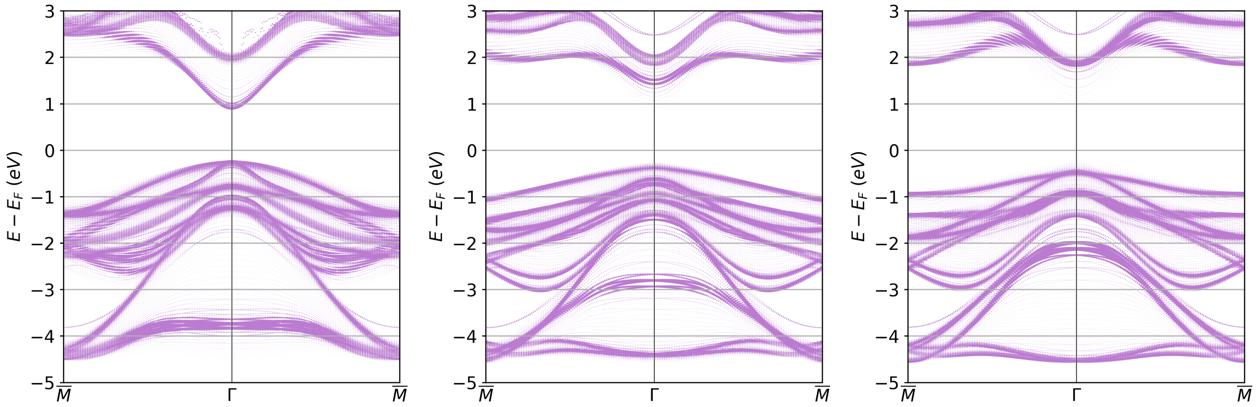

(a) (b) (c)

Figure S8: Dependence on the z-unfolded band structure of CdTe(111) on the choice of k_z: (a) $k_z = 0$ (b) $k_z = 0.3$ and (c) $k_z = 0.5$.

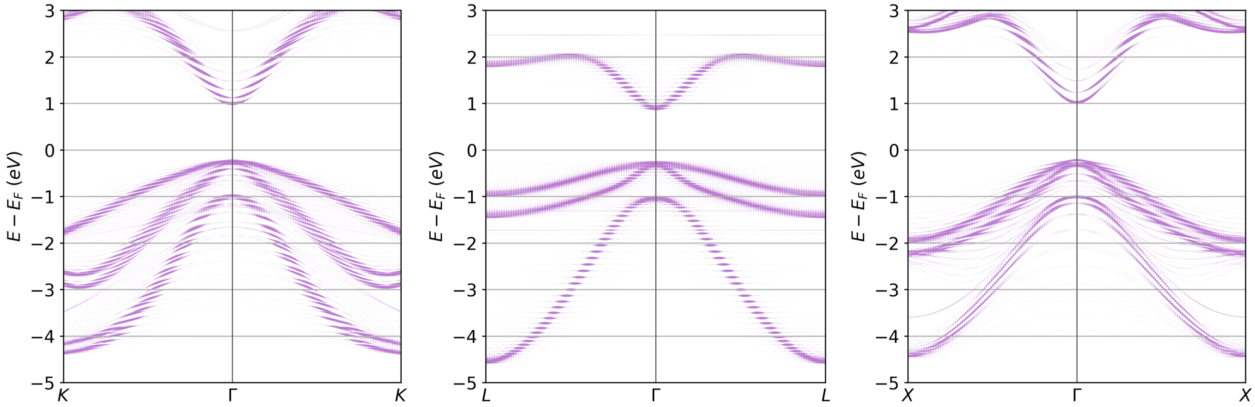

(a) (b) (c)

Figure S9: Some bulk paths with contributions to the z-unfolded band structures of CdTe(111) (shown in Fig. S8): (a) $K - \Gamma - K$, (b) $L - \Gamma - L$, and (c) $X - \Gamma - X$.

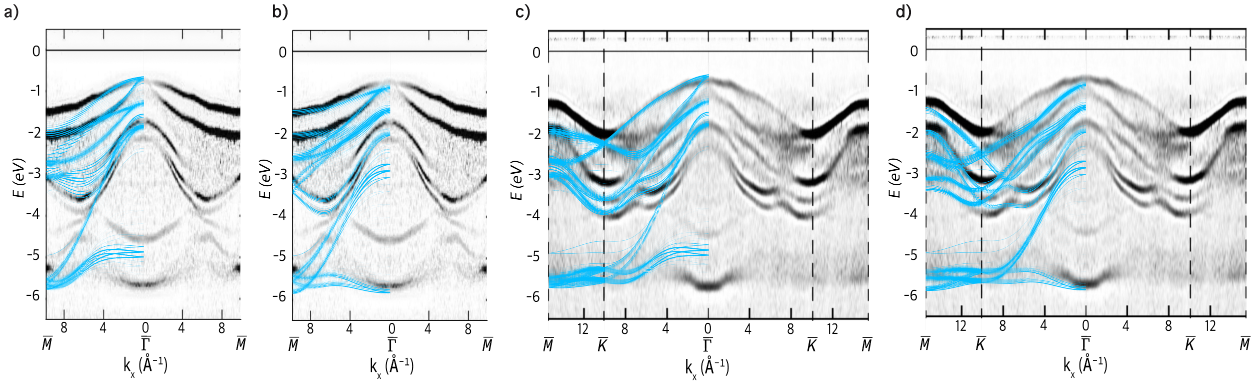

Figure S10: Electronic structure of CdTe: Z-unfolded PBE+U(BO) band structures of CdTe(111) compared with second-derivative map of ARPES data (black and white), adapted with permission from "Spectroscopic studies of CdTe(111) bulk and surface electronic structure" by J. Ren *et al.*, Phys. Rev. B, 91, 235303 (2015); Copyright (2015) by the American Physical Society.[23] Z-unfolded band structures compared to ARPES data along (a), (b) $\overline{\Gamma}-\overline{M}$ and (c), (d) $\overline{\Gamma}-\overline{K}-\overline{M}$. The computed band structures are shown for (a), (c) for $k_z = 0.0$ and (b), (d) $k_z = 0.5$. A shift of -0.25 eV and a stretch factor of 1.22 were applied to the DFT band structures for comparison. This shows that a mix of both k_z values is needed to best reproduce the experimental data.

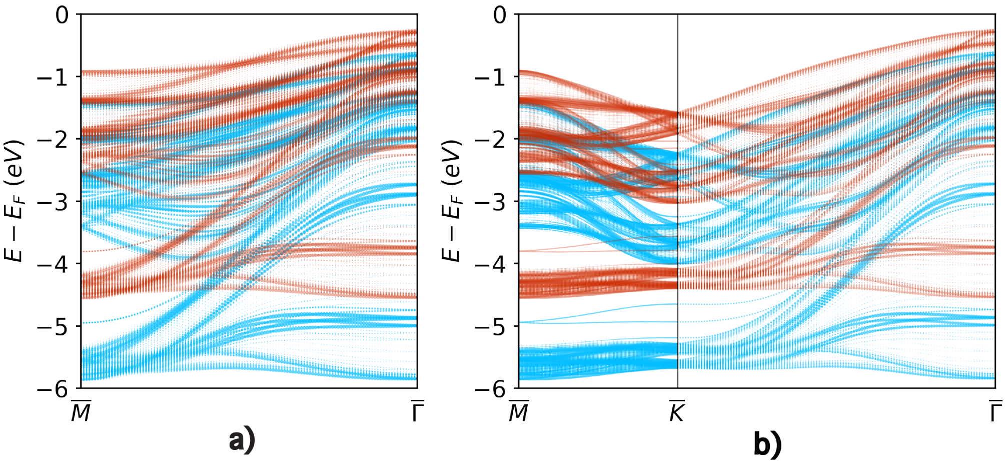

Figure S11: Original z-unfolded band structures (red) compared with the band structures shifted by -0.25 eV and stretched by a factor of 1.22 (light blue). This is shown for CdTe(111) with a mixture of $k_z = 0.0$ and $k_z = 0.5$ for k-paths (a) $\overline{\Gamma}-\overline{M}$ and (b) $\overline{\Gamma}-\overline{K}-\overline{M}$.

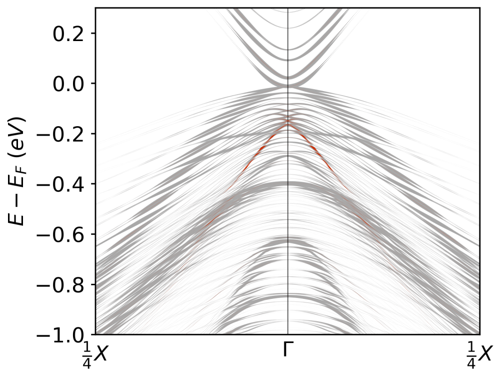

Figure S12: Bulk unfolded band structure of the bilayer InSb/α-Sn interface. The contributions of the top two surface monolayers of α-Sn, which are not in contact with InSb, are colored in red. This shows that the TSS originates from the α-Sn surface on the top of the slab.

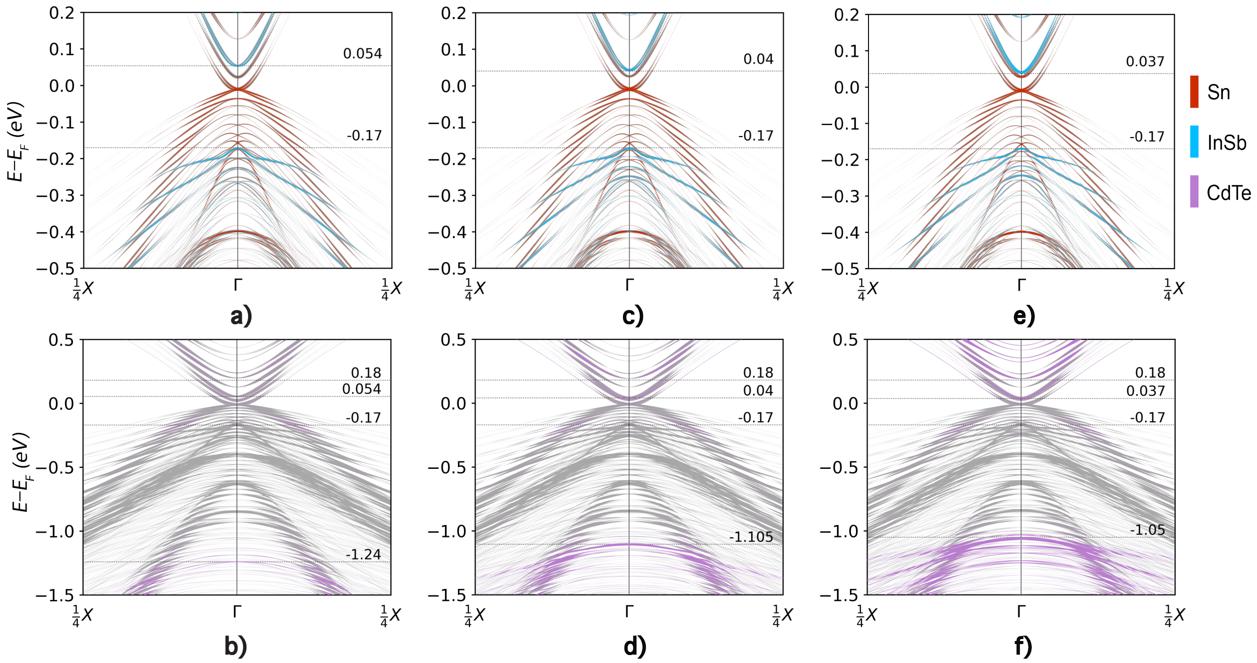

Figure S13: Band alignment of the tri-layer InSb/CdTe/α-Sn interface: Element projected band structures of with (a,b) 6 atomic layers, (c,d) 10 layers, and (e,f) 16 layers of CdTe. The bands originating from α-Sn, InSb, and CdTe are colored in red, light blue, and purple, respectively. Panels (b), (d) and (f) only show the CdTe contributions, due to the different energy scale and to emphasise the CdTe contributions.

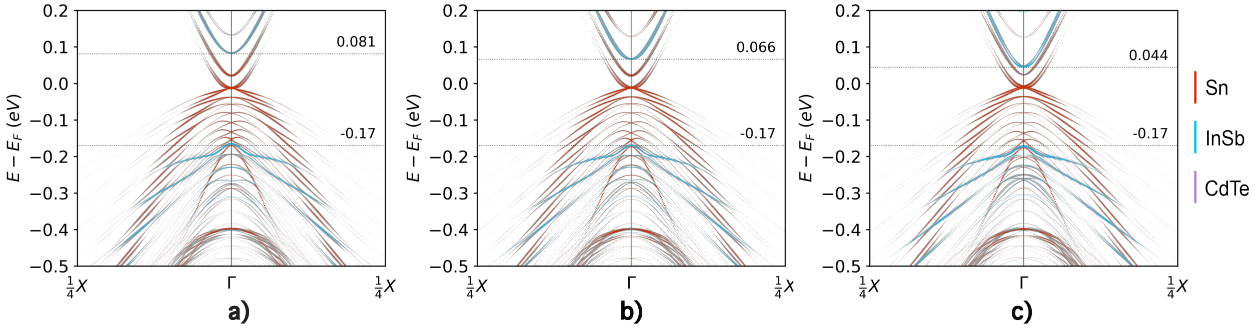

Figure S14: Band alignment of the tri-layer InSb/CdTe/α-Sn interface: Element projected band structures of with (a) 2 atomic layers, (b) 4 layers, and (e) 8 layers of CdTe. The bands originating from α-Sn, InSb, and CdTe are colored in red, light blue, and purple, respectively.

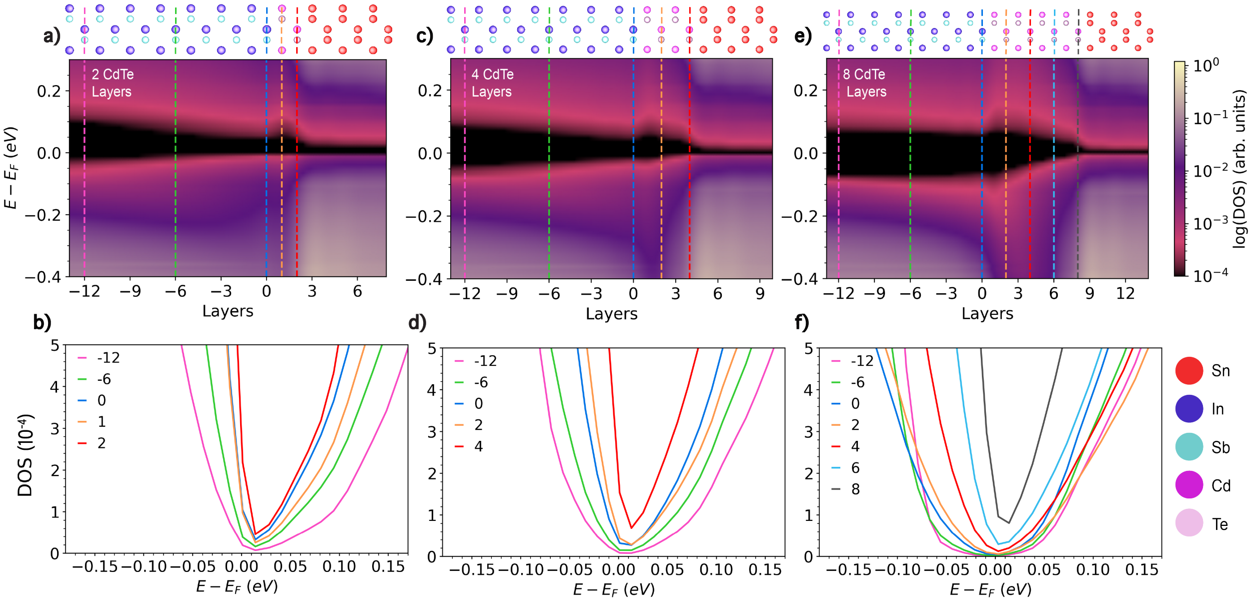

Figure S15: Electronic structure of InSb/CdTe/α-Sn tri-layer interfaces: Density of states as a function of distance from the interface for (a) 2, (c) 4 and (e) 8 CdTe barrier layers. The atomic layers are numbered based on distance from the interface, which is located at zero. Interface structures are illustrated on top. (b), (d), (f) Local density of states for selected layers, indicated by dashed lines in the same colors in panels (a), (c), and (e), respectively.

References

(1) Kresse, G.; Hafner, J. Ab initio molecular dynamics for liquid metals. *Phys. Rev. B* **1993**, *47*, 558–561.

(2) Blöchl, P. E. Projector augmented-wave method. *Phys. Rev. B* **1994**, *50*, 17953–17979.

(3) Kresse, G.; Joubert, D. From ultrasoft pseudopotentials to the projector augmented-wave method. *Phys. Rev. B* **1999**, *59*, 1758–1775.

(4) Perdew, J. P.; Burke, K.; Ernzerhof, M. Generalized Gradient Approximation Made Simple. *Phys. Rev. Lett.* **1996**, *77*, 3865–3868.

(5) Dudarev, S. L.; Botton, G. A.; Savrasov, S. Y.; Humphreys, C. J.; Sutton, A. P. Electron-energy-loss spectra and the structural stability of nickel oxide: An LSDA+U study. *Phys. Rev. B* **1998**, *57*, 1505–1509.

(6) Yu, M.; Yang, S.; Wu, C.; Marom, N. Machine learning the Hubbard U parameter in DFT+U using Bayesian optimization. *npj Computational Materials* **2020**, *6*, 180.

(7) Heyd, J.; Scuseria, G. E.; Ernzerhof, M. Hybrid functionals based on a screened Coulomb potential. *The Journal of Chemical Physics* **2003**, *118*, 8207–8215.

(8) Yang, S.; Schröter, N. B. M.; Strocov, V. N.; Schuwalow, S.; Rajpalk, M.; Ohtani, K.; Krogstrup, P.; Winkler, G. W.; Gukelberger, J.; Gresch, D.; Aeppli, G.; Lutchyn, R. M.; Marom, N. Electronic Structure of InAs and InSb Surfaces: Density Functional Theory and Angle-Resolved Photoemission Spectroscopy. *Advanced Quantum Technologies* **2022**, *5*, 2100033.

(9) Dardzinski, D.; Yu, M.; Moayedpour, S.; Marom, N. Best Practices for First-Principles Simulations of Epitaxial Inorganic Interfaces. *Journal of Physics: Condensed Matter* **2022**,

(10) Küfner, S.; Fitzner, M.; Bechstedt, F. Topological α-Sn surface states versus film thickness and strain. *Phys. Rev. B* **2014**, *90*, 125312.

(11) Shi, Z.; Wang, X.; Xu, C.; Wang, P.; Liu, Y.; Chiang, T.-C. First-principles study of the topological surface states ofα-Sn(111). *Physics Letters A* **2020**, *384*, 126782.

(12) Barfuss, A. et al. Elemental Topological Insulator with Tunable Fermi Level: Strained α-Sn on InSb(001). *Phys. Rev. Lett.* **2013**, *111*, 157205.

(13) Chen, K. H. M.; Lin, K. Y.; Lien, S. W.; Huang, S. W.; Cheng, C. K.; Lin, H. Y.; Hsu, C.-H.; Chang, T.-R.; Cheng, C.-M.; Hong, M.; Kwo, J. Thickness-dependent topological phase transition and Rashba-like preformed topological surface states of α-Sn(001) thin films on InSb(001). *Phys. Rev. B* **2022**, *105*, 075109.

(14) Rogalev, V. A.; Rauch, T. c. v.; Scholz, M. R.; Reis, F.; Dudy, L.; Fleszar, A.; Husanu, M.-A.; Strocov, V. N.; Henk, J.; Mertig, I.; Schäfer, J.; Claessen, R. Double band inversion in α-Sn: Appearance of topological surface states and the role of orbital composition. *Phys. Rev. B* **2017**, *95*, 161117.

(15) Yang, S.; Dardzinski, D.; Hwang, A.; Pikulin, D. I.; Winkler, G. W.; Marom, N. First-principles feasibility assessment of a topological insulator at the InAs/GaSb interface. *Phys. Rev. Materials* **2021**, *5*, 084204.

(16) Wu, Y.; Chen, G.; Zhu, Y.; Yin, W.-J.; Yan, Y.; Al-Jassim, M.; Pennycook, S. J. LDA+U/GGA+U calculations of structural and electronic properties of CdTe: Dependence on the effective U parameter. *Computational Materials Science* **2015**, *98*, 18–23.

(17) Fonthal, G.; Tirado-Mejía, L.; Marín-Hurtado, J.; Ariza-Calderón, H.; Menddoza-Alvarez, J. Temperature dependence of the band gap energy of crystalline CdTe. *Journal of Physics and Chemistry of Solids* **2000**, *61*, 579–583.

(18) Hinuma, Y.; Grüneis, A.; Kresse, G.; Oba, F. Band alignment of semiconductors from density-functional theory and many-body perturbation theory. *Phys. Rev. B* **2014**, *90*, 155405.

(19) Neugebauer, J.; Scheffler, M. Adsorbate-substrate and adsorbate-adsorbate interactions of Na and K adlayers on Al(111). *Phys. Rev. B* **1992**, *46*, 16067–16080.

(20) Yang, S.; Wu, C.; Marom, N. Topological properties of SnSe/EuS and SnTe/CaTe interfaces. *Phys. Rev. Materials* **2020**, *4*, 034203.

(21) Safaei, S.; Galicka, M.; Kacman, P.; Buczko, R. Quantum spin Hall effect in IV-VI topological crystalline insulators. *New Journal of Physics* **2015**, *17*, 063041.

(22) Ozawa, H.; Yamakage, A.; Sato, M.; Tanaka, Y. Topological phase transition in a topological crystalline insulator induced by finite-size effects. *Phys. Rev. B* **2014**, *90*, 045309.

(23) Ren, J.; Fu, L.; Bian, G.; Wong, M.; Wang, T.; Zha, G.; Jie, W.; Miller, T.; Hasan, M. Z.; Chiang, T.-C. Spectroscopic studies of CdTe bulk and surface electronic structure. *Phys. Rev. B* **2015**, *91*, 235303.
